# A 13-year follow-up of Finnish patients with Salla disease

**DOI:** 10.1186/s11689-015-9116-7

**Published:** 2015-07-13

**Authors:** Liisa E. Paavola, Anne M. Remes, Marika J. Harila, Tarja T. Varho, Tapio T. Korhonen, Kari Majamaa

**Affiliations:** Department of Neurology, Oulu University Hospital, P.O. Box 20, 90029 Oulu, Finland; Department of Clinical Medicine, Neurology, University of Oulu, P.O. Box 5000, 90014 Oulu, Finland; Department of Clinical Neurology, University of Oulu, P.O. Box 5000, 90014 Oulu, Finland; Neural Ltd, Center of Neuropsychology, Isokatu 16 B 18, 90100 Oulu, Finland; Department of Neurology, Institute of Clinical Medicine, University of Eastern Finland, P.O. Box 1627, 70211 Kuopio, Finland; Department of Neurology, Kuopio University Hospital, P.O. Box 1777, 70211 Kuopio, Finland; The Neuropediatric Unit of Turku City Welfare Division, P. O. Box 670, 20101 Turku, Finland; Department of Psychology, University of Turku, Turku, 20014 Finland

**Keywords:** Dysmyelination, Follow-up study, Free sialic acid storage, Neurocognitive development, Rare diseases

## Abstract

**Background:**

Salla disease (SD) is a rare lysosomal storage disorder leading to severe intellectual disability. SD belongs to the Finnish disease heritage, and it is caused by mutations in the *SLC17A5* gene. The aim of the study was to investigate the course of neurocognitive features of SD patients in a long-term follow-up.

**Methods:**

Neuropsychological and neurological investigations were carried out on 24 SD patients, aged 16–65 years, 13 years after a similar examination.

**Results:**

The survival analysis showed excess mortality among patients with SD after the age of 30 years. The course of the disease was progressive, but follow-up of SD patients revealed that motor skills improved till the age of 20 years, while mental abilities improved in most patients till 40 years of age. Verbal comprehension skills did not diminish during the follow-up, but productive speech deteriorated because of dyspraxia and dysarthria. Motor deficits were marked. Ataxia was prominent in childhood, but it was replaced by athetotic movements during the teens. Spasticity became more obvious with age especially in severely disabled SD patients.

**Conclusions:**

Younger SD patients performed better in almost every task measuring mental abilities that then seem to remain fairly constant till early sixties. Thus, the results indicate better prognosis in cognitive skills than earlier assumed. There is an apparent decline in motor skills after the age of 20 years. The early neurocognitive development predicts the later course of motor and cognitive development.

## Background

Salla disease (SD; OMIM 604369) is a rare lysosomal storage disease that belongs to the Finnish disease heritage [1–3]. SD is caused by mutations in the *SLC17A5* gene encoding a protein, sialin that is responsible for sialic acid transport across lysosomal membranes and that is required for normal CNS myelination [4]. The prevalence of the major founder mutation, R39C (Salla_FIN_ mutation), is high in the northeast of Finland, where founder effect contributes to the high carrier frequency of 1:100 [5, 6]. Most Finnish SD patients are homozygous for the R39C mutation [6], while a few patients are compound heterozygotes harboring this Salla_FIN_ mutation and another *SLC17A5* mutation. The phenotype of patients with compound heterozygous mutations is more severe than that in patients with homozygous mutations [6, 7]. The severe phenotype is characterized by young age at onset of neurodevelopmental symptoms, motor retardation, cerebral dysmyelination, cerebral and cerebellar atrophy, as well as peripheral nerve involvement [7, 8]. Mutations in the SLC17A5 gene cause also infantile sialic acid storage disease (ISSD; OMIM 269920) that represents the most severe form of lysosomal free sialic acid diseases [9, 10]. The children are severely affected already in utero or the first signs of the disease appear immediately after birth [6, 11, 12], and they usually survive less than 2 years. Mutations found in these patients are different from those in SD patients.

The first symptoms of SD include nystagmus, muscular hypotonia, ataxia, and delayed motor development [2, 7, 13], and they are usually noticed at the age of 3–12 months. All patients become intellectually disabled, but life expectancy is only slightly decreased [2]. Epilepsy is a common symptom. On the basis of disease severity, a conventional and a severe phenotype of SD have been defined [7]. In addition, a few patients have been reported with relatively mild symptoms [7, 14]. Cerebral and cerebellar atrophy, dysmyelination, and corpus callosum hypoplasia are typical for all patients with SD [15–17].

Motor handicap in SD begins to develop in early infancy, and the decline in motor skills is more pronounced than that in cognition after the second decade of life. We have previously carried out a cross-sectional study on neurological findings and neurocognitive profile in 41 Finnish SD patients [7, 18]. Motor disability was severe in these patients, and the characteristic cognitive profile consisted of spatial and visual constructive impairments. Interestingly, the interactive and non-verbal communication skills were quite strong. Here we describe results from a longitudinal study on changes in neurocognitive findings during a 13-year follow-up of 24 Finnish SD patients. We found that the course of the disease was progressive, but follow-up of SD patients revealed that motor skills improved till the age of 20 years, while mental abilities improved in most patients till 40 years of age.

## Methods

### Participants

Forty-one patients with SD were examined in our previous study [18]. Eight patients (six women) had died after this baseline study, six patients (one woman) declined to participate, and three patients (two women) were excluded, because they had not been evaluated with the Bayley Scales of Infant Development (BSID-II) at baseline. The subjects of the follow-up study thus comprised of 24 SD patients (nine women) that were examined 13 years after the baseline study. Nineteen patients were homozygous for the Salla_FIN_ mutation, and five were compound heterozygotes.

The study was approved by the Ethics Committee of Oulu University Hospital. The written informed consent was obtained from the patients and their caregivers.

### Procedure

The patients were examined by the same neuropsychologist and, with two exceptions, by the same neurologist. BSID-II was used to measure the mental and motor development [19] at the follow-up visit (Table 1), and results from BSID-II at the baseline were available for comparison. Developmental age (DA), a measure of a child’s cognitive and motor development expressed in terms of age norms, was also evaluated. The raw scores from the mental and motor tasks of BSID-II were aggregated into respective sum variables, and the achievement of each patient was defined as a percentage of the maximum of the sum variable. The phenotype was further characterized by using the verbal subtests of NEPSY (Children’s Neuropsychological Test Battery) [17, 20], the PANESS test (Physical and Neurological Examination for Soft Signs) [21, 22], the TUG test (Timed Up and Go test) [23], and cerebellar tests [24].

**Table 1:** Methods of neuropsychological evaluation

Abbreviation	Full name of test	Reference	Focus of test
BSID-II	Bayley Scales of Infant Development 2nd ed.	[31]	Motor and mental skills
NEPSY	Children’s Neuropsychological Test Battery (3–6 years)	[20, 24]	Comprehension of instructions
			Oromotor sequences
			Repetition of nonsense words
PANESS	Physical and Neurological Examination for Soft Signs	[22, 32]	Test of corpus callosum functions
Cerebellar tests	Static and dynamic cerebellar tests	[23]	Test of cerebellar functions
TUG test	Timed Up and Go test	[25]	The basic and functional mobility

### Survival analysis

A survival analysis was performed on the 41 patients with SD who had participated in the baseline study. Kaplan-Meier survival analysis and log-rank statistics were used to compare the observed lifetimes of the patients with their life expectancies at birth. The life expectancies were obtained from statistics kept by Statistics Finland [http://www.stat.fi/tup/tilastotietokannat/index_en.html] and were available separately for both sexes and for each year of birth.

### Statistical analysis

BSID-II sum variables were tested for reliability by calculating Cronbach’s alpha. Two groups defined by age (16–30 years, over 30 years) or by gender were compared for differences in neurocognitive development by using Student’s *t* test or Mann–Whitney *U* test, as appropriate. In order to study differences between the developmental ages and the results of BSID-II mental and motor scales after the follow-up period, the paired-sample *t* test was used.

Results in the NEPSY and TUG tests and in cerebellar tasks were used to classify the subjects into three groups. Subjects with no deficits, those with mild to moderate deficits, and those with severe deficits were defined by comparison with the reference values of each test [20, 23–25]. The statistical analyses were conducted using Statistical Package for Social Sciences (SPSS) 20.0 (IBM Corporation, New York, NY, USA) for Windows.

## Results and discussion

### Analysis of the participants in the baseline study and the follow-up

Twenty-four of the 41 SD patients in the baseline study [18] could be recruited to the follow-up study. The participants, the non-participants, and the deceased patients differed at baseline visit in age and mental and motor developmental ages (Table 2). Further analysis revealed that the eight deceased subjects differed significantly from the 24 participants and from the six non-participants (Mann–Whitney *U* test). At baseline, the deceased subjects were older and their mental and motor developmental ages were lower than those of the participants and non-participants. The participants and non-participants did not differ from each other.

**Table 2:** Comparison of participants and non-participants

	Participants	Deceased	Non-participants
*N*	24	8	6
Age, years	19.0 (1–51)	49.5 (28–62)	19.0 (1–62)
Mental DA, months	18.5 (6–41)	8.0 (1–15)	19.0 (8–26)
Motor DA, months	12.0 (2–38)	5.0 (2–11)	12.0 (7–21)

Values are medians (ranges)

*DA* developmental age

**Fig. 1:**
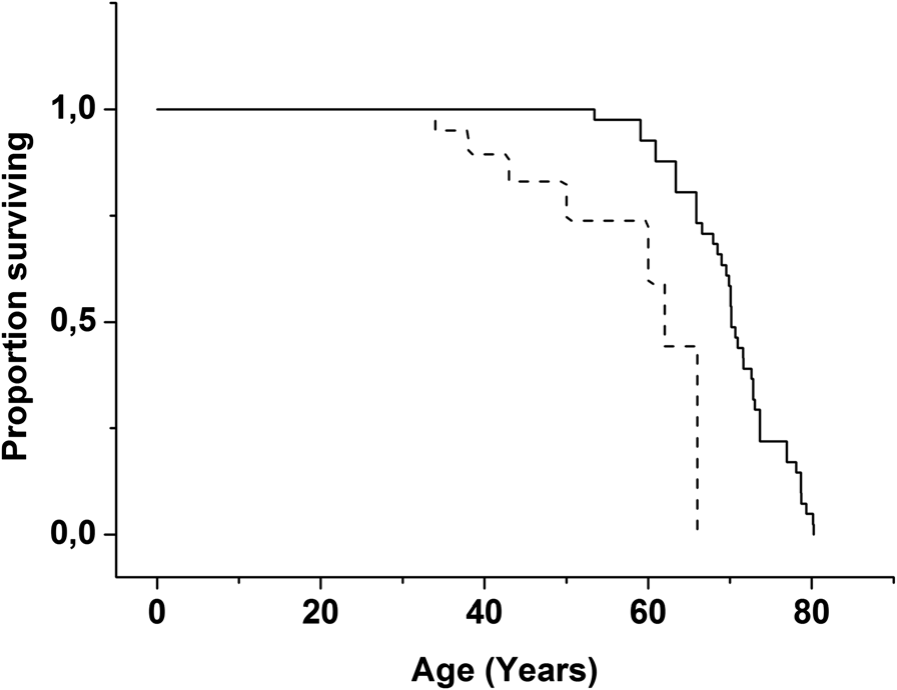
Survival of 41 subjects with SD. Kaplan-Meier survival analysis and log-rank statistics were used to compare the age at death (*dotted line*) and matched life expectancy at birth (*solid line*). Log-rank analysis of the significance of the difference, *p* = 0.00005

The 41 patients with SD who had participated in the baseline study were included in a survival analysis. The analysis showed excess mortality among patients with SD after the age of 30 years (Fig. 1). No difference was noted between the genders (mean survival 57.2 years for women, 59.0 years for men; *p* = 0.88, log-rank analysis). Interestingly, four out of the eight deceased persons had died at the age of 27 years (range, 20–40 years) younger than expected, while four subjects had died 7 years (range, −1–9 years) older than expected. There are no previous studies on mortality of SD patients, and hence, it is not known whether such subgroups are true. Anyhow, these two groups did not differ with respect to mental developmental age (*p* = 0.69, Mann–Whitney *U* test) or motor developmental age (*p* = 0.89) at the baseline visit. All the eight deceased persons had epilepsy during life, but unfortunately, we could not find out whether epilepsy was the cause of death. Patients with SD live longer than those with aspartylglucosaminuria (AGU), another lysosomal storage disorder belonging to the Finnish disease heritage. On average, women with AGU live to 40 years and men to 35 years [26].

### Changes in motor and cognitive skills during the 13-year follow-up

**Fig. 2:**
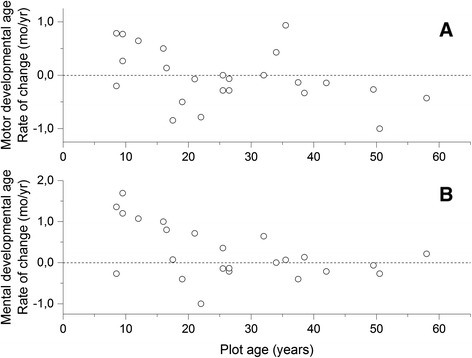
Rate of change in developmental age during the 13-year follow-up. The rate of change in motor (**a**) or mental (**b**) developmental age was calculated per year of follow-up. Data at the baseline visit were obtained from [7]. Values are plotted at the midpoint between the age at baseline and that at the follow-up visit

The median age of the 24 SD patients was 34 years (range, 16–65 years) at the follow-up visit. The rate of change in motor and mental developmental age was calculated for each patient (Fig. 2). Between the baseline study and the follow-up study, the change in motor developmental age (Fig. 2a) was positive till the age of 20 years in most patients, but after that age, motor skills declined. There was no correlation between the rate of change in motor developmental age and the chronological age (Pearson *r* = −0.395, *p* = 0.056). There was an increase in mental developmental age in most patients till their late thirties (Fig. 2b), but beyond that, there was no further change. Indeed, there was an inverse correlation between the rate of change in mental developmental age and the chronological age (Pearson *r* = −0.481, *p* = 0.017). Finally, there was a correlation between the rate of change in mental developmental age and that in motor developmental age (Pearson *r* = 0.685, *p* = 0.0002).

Neurocognitive deficits develop in childhood [7], but the children acquire mental and motor skills till their teens. The baseline study [18] has suggested that several neurodevelopmental periods can be outlined in the clinical progression of SD. The first period includes a normal fetal development as well as the first months after birth, but muscular hypotonia, a delayed motor development, and ataxia then emerge during the first year of life. In the second period, slow development continues until puberty. Severe ataxia is evident in childhood but disappears between ages of 10 and 15 years. The present follow-up study showed that motor development continues till twenties in spite of arising athetosis and spasticity, and mental development continues till thirties. The slowly progressive decline in motor abilities starts after mid-thirties, while mental abilities seem to remain constant till early sixties. Three neurocognitive periods have been described in AGU, another lysosomal storage disease belonging to the Finnish disease heritage [27]. In AGU, a period of positive development in childhood is followed by a gradual loss of skills in the teens and a rapid decline in the twenties, which progression is more severe than that in SD. The phenotype of AGU is less variable than that in SD and, furthermore, resembles more the conventional phenotype of SD [27]. Brain MRI findings of AGU differ from those of SD as the thalami are affected in AGU [28], while corpus callosum hypoplasia, dysmyelination, and cerebral and cerebellar atrophy are constant findings with SD [16].

### Neurological features at the follow-up visit

Neurological evaluation revealed that motor functions were severely affected. Eleven patients were able to walk independently, but all of them had problems in coordination. Seven patients used a walking aid, and six patients (severe phenotype, five; conventional phenotype, one) were non-ambulatory. The non-ambulatory patients were able to make stepping movements and to sit with support. All patients had spastic lower limbs, and the patellar reflexes were abnormally brisk. They also had severe planovalgus, and Achilles reflex was absent. Babinski sign was positive in ten cases.

Twenty-three patients were able to use a partial thumb opposition to grasp an object, and some of them also used the pads of fingertips in grasping or holding a pen. None of the patients could draw a circle or trace designs, but two patients could copy a plus sign.

Dynamic cerebellar tests showed severe deficits in motor sequencing and timing, but none of the patients had ataxia, whereas mild to moderate athetosis was present in 22 patients and two had severe athetosis since the teens. Indeed, ataxia impaired fine motor skills in childhood, but in adults, ataxia was replaced by athetosis. Nystagmus was not observed, but all patients had a moderate to severe strabismus.

Twelve patients had a history of epileptic seizures, but EEG was available only from four patients. Based on case histories, both primary and secondary generalized epilepsies were assumed. Eight patients were on monotherapy, while the remaining four were on polytherapy. In addition, three patients presented with startle-type reaction to auditory stimulus. The median age at onset of epilepsy was 29 years, and the patients with epilepsy were significantly older than those without (*p* = 0.007). An analysis of 121 patients with AGU has shown that 28 % of the adults but only 2 % of the children have epileptic seizures [29, 30]. These figures suggest that SD and AGU differ from each other in the onset of epilepsy.

Comparison of the clinical features at baseline [7, 18] and at the follow-up visit suggested that spasticity becomes more obvious with age especially in severely disabled SD patients. Severe motor handicap is typical for the conventional phenotype as well as the severe phenotype. One third of the affected children examined at baseline had learned to walk, and in the follow-up examination, a similar proportion of patients were ambulatory. Dysmyelination of the CNS probably explains the decline in motor and mental skills. The dysmyelination is expressed as homogeneous or periventricular white matter disease in most patients and as thin corpus callosum in all patients [16].

### Neurocognitive functions at the follow-up visit

The receptive verbal skills were better than speech production (*p* = 0.003; related samples Wilcoxon signed-rank test), e.g., the patients were able to perform tasks that demand comprehension of instructions. All the patients were able to vocalize a single sound, and the patients with the conventional phenotype were able to use at least two words appropriately, while patients with the severe phenotype could not imitate words.

Concentration skills varied markedly among the patients. Compared to the results of healthy children of the age of 3.5 years described in the test manual of BSID-II, visual tracking and visual attention tasks were performed well, as well as tasks that demanded eye-hand coordination (e.g., use of a spoon or comb). Tasks related to visuospatial reasoning revealed cognitive deficits in all patients and visuomotor performance was slow. All patients recognized familiar faces and voices and responded to a smile and showed emotional states either verbally or non-verbally. Talkative patients were able to remember songs and phraseologies and to learn new ones. Most of the patients who could speak had dysarthria or dyspraxia, but none of the patients had aphasia. The caregivers described that the patients learned daily routines and could keep short instructions in mind. Patients could not perform the subtests of PANESS. Performance in the dynamic cerebellar tests, verbal tasks of NEPSY, and the TUG test is described in Table 3.

**Table 3:** Frequency and severity of deficits in language and fine motor skills among 24 patients with SD

Test	No. of deficits (*n*)	Mild or moderate deficit (*n*)	Severe deficit (*n*)
NEPSY			
Comprehension of instructions	14	3	7
Oromotor sequences	0	1	23
Repetition of nonsense words	2	1	21
Dynamic cerebellar tests			
Finger to thumb	1	1	22
Toe tapping	8	4	12
Pegboard task	0	9	15
Bead threading	3	4	17
TUG test	2	8	14

The median age of 30 years was used to define two groups. The younger group performed better in almost every task of the mental scale. Significant differences were found in constructive skills (*p* = 0.026), basic counting (*p* = 0.016), and immediate visual recognition (*p* = 0.026). No differences were detected in visual attention and interactive skills between the two groups.

**Fig. 3:**
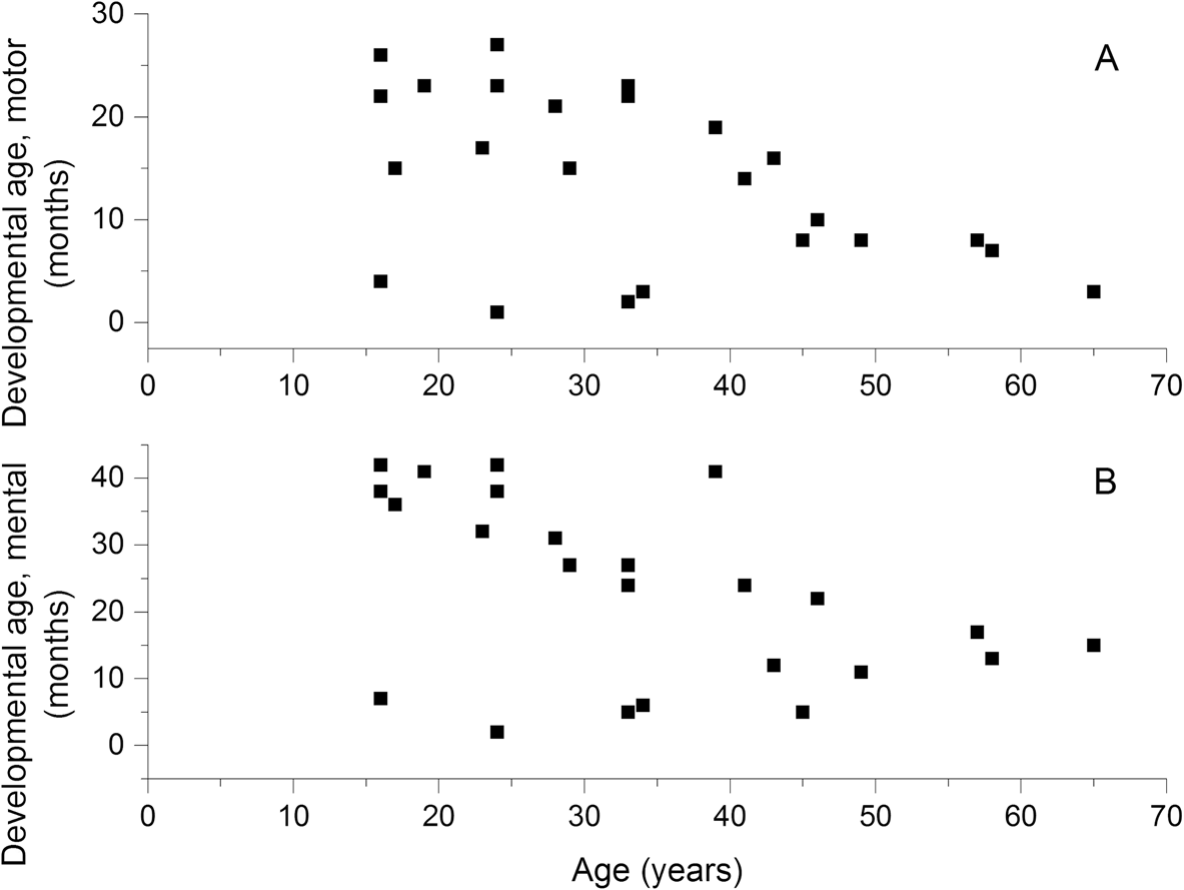
Developmental age as a function of chronological age in 24 patients with SD. Developmental age was determined by using the motor tasks (**a**) or the mental tasks (**b**) of BSID-II

The maximal motor developmental age was 27 months, and the maximal mental developmental age was 42 months among the 24 SD patients. The developmental ages of four subjects (age range, 16–34 years) were significantly lower than those of other patients with a similar age. These four patients presented with the severe phenotype of SD, and they were compound heterozygotes harboring the R39C mutation only in one allele. There was a significant inverse correlation between the motor developmental age and the chronological age (Fig. 3a) and between the mental developmental age and chronological age (Fig. 3b). Motor and mental developmental ages at the follow-up visit correlated well with each other (Pearson correlation coefficient *r* = 0.88, *p* = 0.001), and this was the case also for the data obtained at the baseline visit. The motor developmental age at the follow-up visit was dependent on that at the baseline visit, but not on age, sex, or baseline mental developmental age. In a similar fashion, the mental developmental age at the follow-up visit was dependent on that at the baseline visit, but not on age, sex, or motor developmental age at baseline.

## Conclusions

In this study, we examined the course of clinical features in patients with SD in a 13-year follow-up study. The results of this follow-up study suggest better prognosis in cognitive skills than previous cross-sectional studies. Motor development continues till twenties and mental development till thirties. The slowly progressive decline in motor abilities starts after mid-thirties, while mental abilities seem to remain constant till early sixties. The motor handicap is severe, whereas the cognitive skills related to verbal comprehension and interactive skills do not deteriorate in adulthood. The early neurocognitive development predicts the later course of motor and cognitive development.
